# Machine learning approach as an early warning system to prevent foodborne *Salmonella* outbreaks in northwestern Italy

**DOI:** 10.1186/s13567-024-01323-9

**Published:** 2024-06-05

**Authors:** Aitor Garcia-Vozmediano, Cristiana Maurella, Leonardo A. Ceballos, Elisabetta Crescio, Rosa Meo, Walter Martelli, Monica Pitti, Daniela Lombardi, Daniela Meloni, Chiara Pasqualini, Giuseppe Ru

**Affiliations:** 1 Istituto Zooprofilattico Sperimentale del Piemonte, Liguria e Valle d’Aosta, Via Bologna 148, 10154 Turin, Italy; 2 Tecnológico de Monterrey, Av. Eugenio Garza Sada 2501 Sur, Tecnológico, 64849 Monterrey, N.L. México; 3 Department of Computer Science, University of Turin, Corso Svizzera 185, 10149 Turin, Italy; 4 Piedmont Regional Service for the Epidemiology of Infectious Diseases (SeREMI), Via Venezia 6, 15121 Alessandria, Italy

**Keywords:** Supervised learning, decision tree algorithms, disease surveillance, food products, salmonellosis, transdisciplinarity

## Abstract

Salmonellosis, one of the most common foodborne infections in Europe, is monitored by food safety surveillance programmes, resulting in the generation of extensive databases. By leveraging tree-based machine learning (ML) algorithms, we exploited data from food safety audits to predict spatiotemporal patterns of salmonellosis in northwestern Italy. Data on human cases confirmed in 2015–2018 (*n* = 1969) and food surveillance data collected in 2014–2018 were used to develop ML algorithms. We integrated the monthly municipal human incidence with 27 potential predictors, including the observed prevalence of *Salmonella* in food. We applied the tree regression, random forest and gradient boosting algorithms considering different scenarios and evaluated their predictivity in terms of the mean absolute percentage error (MAPE) and R^2^. Using a similar dataset from the year 2019, spatiotemporal predictions and their relative sensitivities and specificities were obtained. Random forest and gradient boosting (R^2^ = 0.55, MAPE = 7.5%) outperformed the tree regression algorithm (R^2^ = 0.42, MAPE = 8.8%). *Salmonella* prevalence in food; spatial features; and monitoring efforts in ready-to-eat milk, fruits and vegetables, and pig meat products contributed the most to the models’ predictivity, reducing the variance by 90.5%. Conversely, the number of positive samples obtained for specific food matrices minimally influenced the predictions (2.9%). Spatiotemporal predictions for 2019 showed sensitivity and specificity levels of 46.5% (due to the lack of some infection hotspots) and 78.5%, respectively. This study demonstrates the added value of integrating data from human and veterinary health services to develop predictive models of human salmonellosis occurrence, providing early warnings useful for mitigating foodborne disease impacts on public health.

**Supplementary Information:**

The online version contains supplementary material available at 10.1186/s13567-024-01323-9.

## Introduction

The One Health concept has increasingly gained strength in recent years, stressing the need for a transdisciplinary approach to addressing public health concerns. Foodborne pathogens are globally acknowledged as among the most important health priorities due to their direct impact on public health, the economy and society [1]. In 2021, European Union (EU) Member States reported 4005 foodborne outbreaks, resulting in 32 543 cases of illness, 2495 hospitalisations and 31 deaths. Domestic and public settings, including catering, restaurants and canteens, were the main point sources of exposure to contaminated food for most of the cases. Nontyphoidal *Salmonella* was the second most important enteric pathogen involved in foodborne infections, accounting for 19.3% of all outbreaks. Eggs and egg products, mixed foods, bakery products, vegetables and juices and other products thereof were among the main food sources of *Salmonella* infection, although composite or multiingredient foods were generally responsible for the greatest number of illnesses [2].

In Europe, *Salmonella* surveillance is governed by Directive 2003/99/EC [3], which obliges EU Member States to collect relevant information on pathogens, antimicrobial resistance and foodborne outbreaks. In parallel, its surveillance in humans is performed by the network for the epidemiological surveillance and control of communicable diseases [4], to which EU Member States adhere. This feeds the metadata-driven platform (TESSy) of the European Centre for Disease Prevention and Control [5]. In Italy, disease surveillance benefits from standardised and functional communication channels that have been in place for a long time. The implemented animal health surveillance programmes are coordinated at the national level, ensuring an even distribution of activities throughout the territory. In northwestern Italy, a central laboratory (i.e., the *Istituto Zooprofilattico Sperimentale del Piemonte*, *Liguria e Valle D’Aosta*, *IZSPLVA*) manages the data generated by veterinary activities in the field and transmits them to competent regional and national authorities. As a result, the *IZSPLVA* maintains large databases, and the validity of these databases has been verified over the years. In parallel, human disease surveillance data are collected and collated by the Piedmont Regional Service for the Epidemiology of Infectious Diseases (SeREMI). Surveillance activities for certain zoonoses, including salmonellosis, are coordinated at the regional level for both animals and humans. However, current data on zoonoses generated by laboratories or medical and veterinary health services often result in very large and heterogeneous databases that rarely communicate with each other or have minimal opportunities for interconnection [6]. The opportunity to use such datasets (“*big data*”) with a multidisciplinary approach is often overlooked. However, available data analysis methods enable the processing and/or transformation of data with high epidemiological value and great significance in terms of health prevention.

Artificial intelligence techniques, such as machine learning (ML), have been widely exploited in medical and public health research [7–9] due to the potential advantages this discipline offers in terms of health protection and promotion while increasing the efficiency of health services [10]. These tools facilitate the extraction of the underlying information contained in big data, enabling the discovery of otherwise invisible patterns that are valuable for public health and epidemiological research [11–14]. When the emphasis is on prediction rather than inference (which falls under the classical domain of statistics), ML algorithms have displayed pronounced success. In the field of foodborne diseases, ML techniques have been employed to forecast the number of incident cases caused by selected foodborne pathogens [15–18], to identify food attributions or the causative agent responsible for human outbreaks [19–21] and to evaluate the spatial risk of human outbreaks [22]. The identification of common spatial and temporal features in food and human data using ML may pave the way for the early detection of warning signals and the adoption of effective prevention strategies. Despite its potential, the current data collection methods for both veterinary and human epidemiological surveillance are usually separate and often neglect data integration.

Therefore, we aimed to demonstrate the added value of integrating data on the occurrence of salmonellosis in humans and food products in the Piedmont region of northwestern Italy. In particular, we assessed the potential of food data generated by regional food safety surveillance activities to predict spatiotemporal patterns of emerging human infections by applying different tree-based ML algorithms. The data generated by both surveillance systems from 2014 to 2018 were used to develop optimal prediction models, whereas the food surveillance data from 2019 were used to predict the incidence of human salmonellosis in the same year.

## Materials and methods

### Data sources and processing

Data on *Salmonella* infections were obtained from different information databases and retrieved separately from each of the consulted information systems. We collected all the cases of human infection reported in the Piedmont region between January 1^st^, 2015, and December 31^st^, 2019. The computerised SeREMI system, called “*Sistema Informatizzato Malattie Infettive*” (SIMI, [23]), provided the data on human infections. The SIMI collects all probable and/or confirmed cases of infectious aetiology reported by physicians at the regional level. These data were extracted using the GeMInI web-based database [24], with the inclusion criteria based on the Code 003 of the International Classification of Diseases (ICD-9). This code identifies *Salmonella* infections and excludes those caused by *S. typhi* and *S. paratyphi*. To ensure comprehensive case detection, we additionally collected human salmonellosis data from the EnterNet Italia platform [25]. This portal records information concerning enteric pathogens involved in confirmed clinical cases at the national level. From EnterNet, we extracted all the records related to Piedmont’s *Salmonella* infections during the specified time period. The obtained human datasets were integrated by matching records based on birth date, sex, location of symptom onset, or, in the absence of this information, place of residence. This data integration provides added value by improving the characterisation of the health issue and potentially identifying human cases that may have been missed by the SeREMI system.

Data on food products were obtained from the *IZSPLVA* laboratory information system called SIGLA, which records all institutional activities related to animal research and routine laboratory testing. We retrieved the data using general PL/SQL queries, which is a common method used at the *IZSPLVA* for data analysis and routine reporting activities. The resulting dataset contained nonaggregated records, including details of sample collections, such as geographical origin, animal species, type of laboratory analyses performed, and results. Among the features retrieved from the SIGLA system, no variable was dedicated to uniquely identifying specific diseases. This required several steps of accurate data processing before the data were ready for use (Additional file 1). Our inclusion criteria focused on food products collected in the Piedmont region between July 1^st^, 2014, and December 31^st^, 2019. We selected specific laboratory tests for *Salmonella* detection or untargeted laboratory tests, such as bacterial isolation, in which *Salmonella* spp. were identified. The resulting dataset was then checked for duplicates and cleaned, giving priority to confirmed positive results when multiple laboratory tests on the same sample yielded contrasting results.

Differences in the types of data collected between the food and human datasets led to the use of different measures of disease frequency prior to data integration. The human databases contained only positive/confirmed disease cases, allowing the calculation of disease incidence based on the resident population. By contrast, the animal/food database included both positive and negative results for pathogen detection, allowing the prevalence of infection in food products to be estimated.

In addition, we chose to use the open-source dataset provided by the Italian National Institute of Statistics (ISTAT, [26]) to compile the demographic and spatial data of the Italian territory. These data were needed to calculate the denominators of the resident population and to integrate the human and food datasets.

### The dataset

We initiated the construction of the working dataset by focusing on human data collected from 2015 to 2018. These data were aggregated at the municipality level, calculating the monthly incidence rates of *Salmonella* infections (shown as the dependent variable, *H_INC*), and standardised by sex and age. Consequently, the epidemiological unit of the dataset consisted of a specific combination of a municipality where salmonellosis cases arose and a one-month interval (*H_MONTH*). Next, we assigned a value of *Salmonella* prevalence detected in food products (the predictive variable, *F_PREV*) for each epidemiological unit. This was determined by considering a hypothetical exposure area (the potential area of food supply) and a time lag that took into account the municipalities where consumers were most likely to purchase food products, the incubation period of the disease (from pathogen exposure to illness onset), and the time elapsed between the onset of symptoms, disease case detection and notification of health authorities.

**Figure 1:**
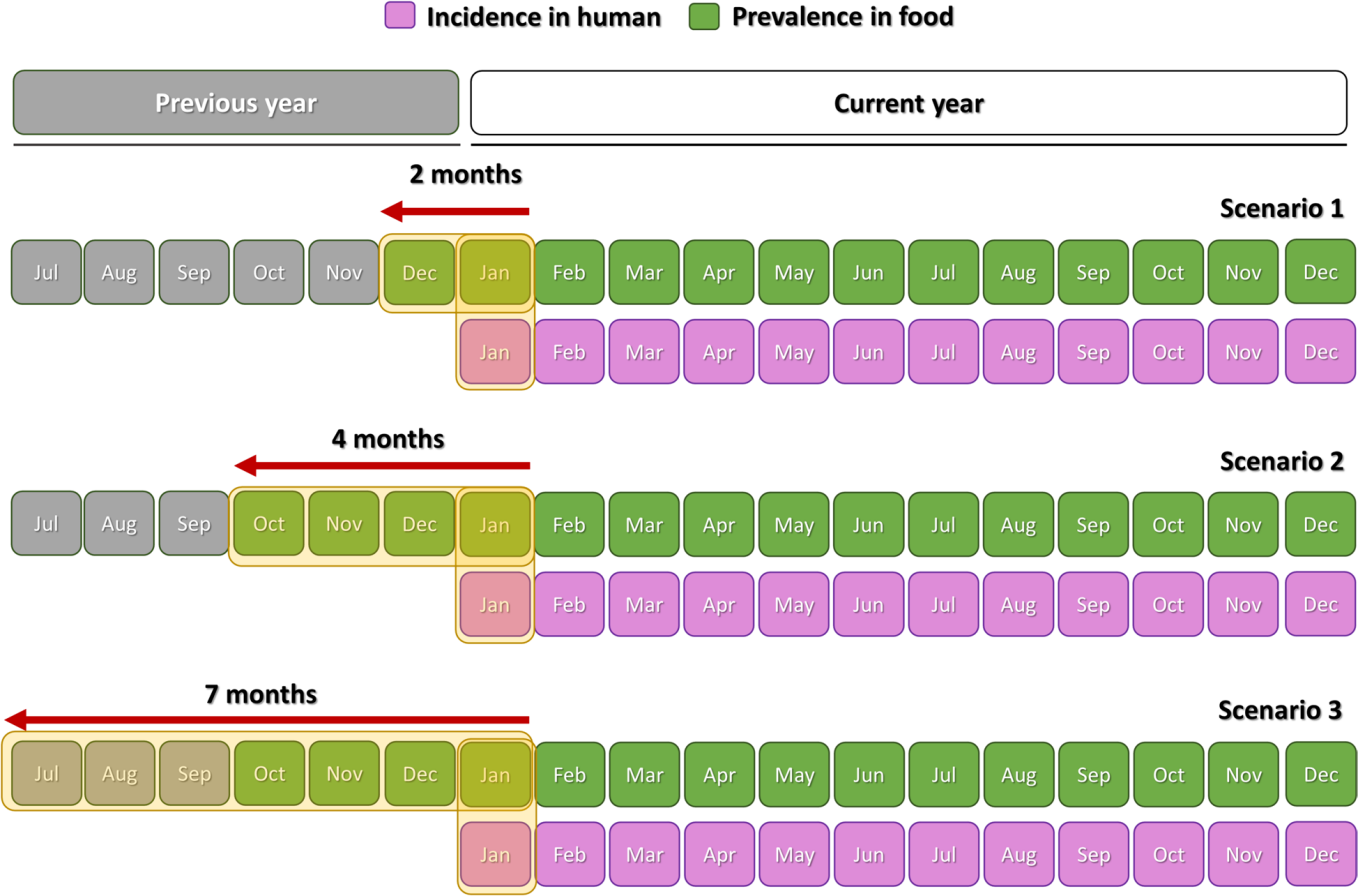
**Time intervals established for estimating the prevalence of** ***Salmonella*** **contamination in food products from the hypothetical food supply areas (areas of exposure) within each spatial–temporal scenario.**

Foodborne disease outbreaks generally involve contamination from a single point source in localised areas, and the infection only occasionally spreads through the supply chain to geographically distant locations [27]. Hence, we determined the potential exposure area based on the average size of the municipalities as well as the distance between them. The calculation of *F_PREV*, reflecting the proportion of positive food samples out of the total tested, was performed in the area encompassing the municipality where human cases emerged and among their nearest neighbours. Both the incubation period and the notification process were considered when accounting for the time lag between infection and detection as a case in the information systems. All infectious diseases exhibit an incubation period. In nontyphoidal *Salmonella* infections, the typical reported duration of infection is between 6 and 72 h [28]. Nonetheless, longer incubation periods of 9 to 16 days have been recorded [29, 30]. Additionally, due to the delay associated with case identification, confirmation and subsequent reporting to health authorities, the time lag can be quite long [31]. To address these complexities, we devised three different temporal scenarios by linking the *H_INC* recorded in a specific municipality and month with the *F_PREV* determined in the exposure area during three predefined time lags (i.e., lags of two months, four months and seven months) from the emergence of human cases (Figure 1). Therefore, taking into account the 2-month time lag, the *F_PREV* expressed for *H_INC* occurring in March 2015 was calculated by considering all the food products tested and exhibiting in positive results from an exposure area during February and March 2015.

We excluded municipalities where no human salmonellosis cases were recorded by health services or where no food product monitoring was conducted during the specific time interval. Each record was enriched with additional explanatory features intended to offer a more comprehensive understanding of the observed human incidence (Table 1). These features included spatial characteristics such as the centroid coordinates (*DDcoordY* and *DDcoordX*) of the municipalities where human cases emerged; the total surface area of the exposure area (*SUPKM2*), expressed in km^2^; the quantity of food samples with positive results; and the total number of laboratory assays conducted for each exposure area across 11 different food categories.

**Table 1:** **Description of the explanatory features used as inputs for the ML models in the study**

Predictors	Name of the features	Description
Time		
Month	*HMONTH*	Actual month in which incident cases emerged or may have emerged
Spatial		
Longitude	*DDcoordX*	Centroid coordinates of municipalities in which human cases have emerged
Latitude	*DDcoordY*	
Area of exposure	*SUPKM2*	Area of pathogen exposure, expressed in km^2^
Food		
Food prevalence	*F_PREV*	Prevalence of *Salmonella* contamination in food products
Food categories		Type of foods tested within the food safety surveillance

	Name of the food category	N. of tests	N. of positive samples	Note
1	Eggs	*EGG*_*t*_	*EGG*_*p*_	Including their products and derivatives
2	Milk	*MILK*_*t*_	*MILK*_*p*_	
3	Cereal-based products and legumes	*CERELEGUM*_*t*_	*CERELEGUM*_*p*_	Cereals, flour, pasta, dough, legumes
4	Fruits and vegetables	*FRUIT*_*t*_	*FRUIT*_*p*_	Fresh and frozen products and vegetable sauces
5	Bakery products	*BAKERY*_*t*_	*BAKERY*_*p*_	Creams, fruit jams, syrup and candied fruits, cookies
6	Seafood	*FISH*_*t*_	*FISH*_*p*_	Fresh, frozen and canned products and fish-based sauces
7	Poultry meat	*POULTRY*_*t*_	*POULTRY*_*p*_	Fresh, frozen, cured meats and processed meat products, including animal byproducts
8	Beef	*BEEF*_*t*_	*BEEF*_*p*_	
9	Pig meat	*PIG*_*t*_	*PIG*_*p*_	
10	Various meats	*V_MEAT*_*t*_	*V_MEAT*_*p*_	Fresh, frozen, cured and minced meats and processed products, including animal byproducts, from different animal species (e.g., horse, lamb, goat)
11	Ready-to-eat foods	*READY*_*t*_	*READY*_*p*_	Products for direct consumption without the need for cooking or other processing

The entire dataset consisted of 220 observations, all of which contained complete data on both the dependent variable (*H_INC*) and the 27 predictors.

A significant challenge faced during this study was to find sufficient data on food products to develop parameter estimates. After integrating the data, only 220 out of the initial 1377 records provided complete information. To enhance the performance of the ML algorithms, we therefore simulated a scenario where we had *Salmonella* prevalence data for food products available for all the epidemiological units and time lags under investigation. This methodology allowed us to develop three ML algorithms that were later used to pursue our objectives and evaluate their suitability for our dataset. To achieve this, we applied Laplace smoothing to the initial tests conducted and positive outcome tallies [32]. This procedure assumed the requirement of further tests or samplings ($\alpha$) for detecting pathogens in foodstuffs per sampling area and lag time, irrespective of the food category. By including a minimum ‘corrected’ prevalence (${p}_{c}$), we could integrate the previously excluded records that lacked prevalence information. Here, ${p}_{c}$ was calculated as follows:

$${p}_{c}=\frac{{n}_{i} +\alpha \left(\frac{\sum_{i=1}^{n}{x}_{i}}{n}\right) }{n+\alpha }$$

where.

${n}_{i}$ represents the observed number of *Salmonella*-positive food products tested;

$\alpha$ represents the hypothetical number of additional tests needed;

$\left(\frac{\sum_{i=1}^{n}{x}_{i}}{n}\right)$ denotes the mean prevalence observed in food products within a given sampling area and time lag; and.

$n$ represents the actual number of tests conducted in a given sampling area and period of time.

A set of $\alpha$-values was defined based on the municipality’s population size and the specified time lag for each scenario (Additional file 2, Sect. 4, Table S2). For this purpose, we classified the municipalities in the Piedmont region into five distinct groups: (1) those with a population ≤ 5000 inhabitants, (2) those with a population between 5001 and 9999, (3) those with a population between 10 000 and 19 999, (4) those with a population between 20 000 and 29 999, and (5) those with a population ≥ 30 000 inhabitants. The parameters estimated for the model using this approach, along with the temporal scenario, which exhibited the best fit and the lowest mean absolute percentage error (MAPE), were considered the best results in the simulated modelling performance assessment.

We compiled a final dataset containing exclusively data from the 2019 food safety surveillance. This was achieved by following the aforementioned procedure and adhering to the optimal temporal scenario. The dataset that resulted contains a total of 1035 observations, each equipped with complete information on the 27 explanatory features that are outlined in Table 1. Considering our aim to predict the emergence of human salmonellosis, this dataset, which was not used for model testing purposes, was treated as unlabelled data. This means that information on human salmonellosis, which was treated separately and later used for comparisons with the models’ predictions, was lacking.

### Statistical analyses

All data management, preprocessing and analyses were performed using Stata 17 [33], whereas graphical representation of the results was obtained by using R (version 4.2.2) and QGIS3 (version 3.4 Madeira) software. We calculated the proportion of human salmonellosis detected by the human health system as well as the prevalence of *Salmonella* in food products and 95% exact binomial confidence intervals (CIs). Initially, we evaluated the potential relationship between *H_INC* and *F_PREV* (Additional file 2, Sect. 1, Figure S4). For this purpose, we utilised data from the entire study period (2014–2019), considering the different designed time scenarios. A log–log linear regression model was used to fit the natural logarithm transformation of both variables, with *H_INC* representing the dependent variable and *F_PREV* serving as the explanatory variable.

A range of epidemiological studies on foodborne diseases have employed tree-based ML algorithms [15, 17, 19–21]. We ran and fitted tree regression (*TR*), random forest (*RM*) and gradient boosting (*GB*) algorithms using the recently developed r_ml_stata_cv command [34]. This command makes use of the Python Scikit-learn API for both cross-validation and outcome prediction. To determine the model with the best performance, we conducted five-fold cross-validation. This method randomly splits the training dataset into five equal-size portions, called *folds.* Here, four folds were used for model training (*in-sample*), and the remaining fold was used to estimate model performance (*out-of-sample*). This procedure is repeated until all five folds have been used for testing five distinct models trained on the remaining folds, each using unique and separate training and testing folds. The prediction error estimates are obtained by averaging all *out–of–sample* mean square errors obtained fold–by–fold. *K*-fold cross-validation also provides an estimation of the true test error (i.e., mean absolute percentage error, MAPE), which enables us to evaluate the uncertainty of the best-optimised model. The tuning of the hyperparameters of each ML algorithm was modified from the default values based on the grid search strategy [35] using the values reported in Table 2 to optimise algorithm performance.

**Table 2:** **The parameters used in tree regression (*TR*), random forest (*RF*) and gradient boosting (*GB*) ML algorithms**

ML algorithm	Parameter	Real prevalence	Simulated prevalence
*TR*	Maximum tree depth	25	20
*RF*	Maximum tree depth	25	20
	Max. no. of splitting features	27	5
	Max. no. of bootstrapped trees	50–250	50–250
*GB*	Maximum tree depth	25	20
	Learning rate	0.1–0.3	0.1–0.3
	Number of sequential trees	50–250	50–250

To develop our ML algorithms, we used two collections of data: human data from 2015 to 2018 and food data from 2014 to 2018. These data were treated as both training and test datasets by randomly selecting data from the original dataset at a 7:3 ratio. The scenario that yielded the best model fit and precision was selected for the prediction of human salmonellosis in 2019. The unlabelled dataset for this task was the 2019 food safety surveillance data, which was employed to evaluate the generalisation performance of the predictive model that had been trained on data from 2015 to 2018 (Additional file 2, Sects. 3 and 5). The predictions obtained were then compared with the incidence of human salmonellosis recorded in 2019 by human health surveillance systems. In addition, we evaluated the sensitivity and specificity of the models when used to predict the observed disease occurrence status (in terms of the presence or absence of at least one case) of each municipality.

## Results

**Figure 2:**
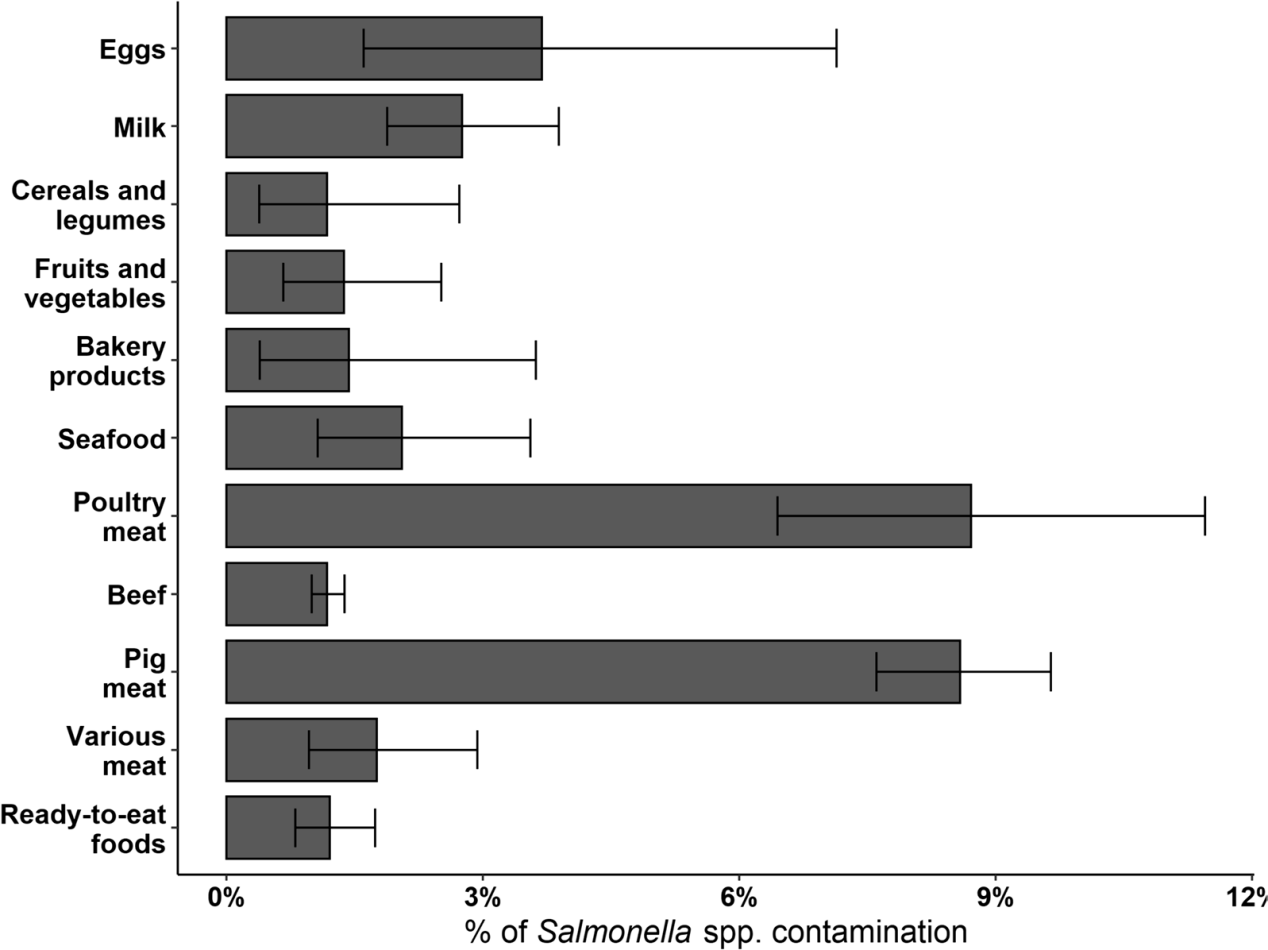
**Prevalence and 95% CIs of** ***Salmonella*** **spp. contamination in food products, the Piedmont region 2014–2019.**

The regional health services recorded 2560 *Salmonella* infections in the human population from 2015 to 2019, resulting in an average incidence rate of 5.8 per 10 000 person-years. The infections were distributed among all age and sex strata, with the youngest population displaying the highest infection rates (Table 3). We noted differences in the retrieval of disease occurrence records depending on which human data sources were used. Specifically, only 36.5% (*n* = 935) of the cases were commonly shared between both human databases, whereas the remaining 22.3% (*n* = 572) and 41.1% (*n* = 1053) of the cases specifically originated from the SeREMI and Enternet databases, respectively. Regarding food safety surveillance, the system revealed a *Salmonella* spp. prevalence of 2.5% (95% CI 2.3–2.7) in food products monitored between 2014 and 2019. The highest levels of *Salmonella* spp. contamination were found in poultry and swine meat products, with other food categories not exceeding a prevalence of 3.7% (Figure 2).

**Table 3:** **Salmonellosis incidence rates (IRs) in the Piedmont region from 2015 to 2019**

		Males		Females		
Age strata	n	Average population	IR_m_	n	Average population	IR_f_
0–9	594	187 047.4	31.8	526	176 086.6	29.9
10–24	160	299 289.4	5.3	120	279 169	4.3
25–49	95	701 997.4	1.4	122	698 011.4	1.7
50–74	243	707 284.2	3.4	197	755 683.4	2.6
≥ 75	186	227 447.6	8.2	188	348 495.8	5.4

Incidence rates are presented as cases per 10 000 person-years and stratified by sex and age. A total of 129 cases of salmonellosis were omitted from this table because both sex and age data were unavailable. IR_m_ represents the incidence rate for males, and IR_f_ represents the incidence rate for females.

**Figure 3:**
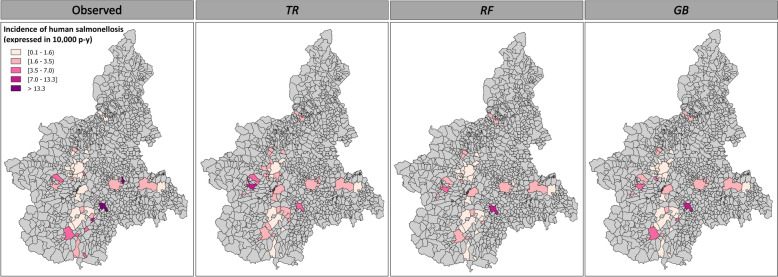
**Observed and predicted incidence rates of human salmonellosis recorded across 39 municipalities in the Piedmont region during 2019.** **These data provide a partial representation of the actual distribution of human cases, as they only include the epidemiological units (a combination of time intervals and municipalities) for which data on both human salmonellosis (*H_INC*) and** ***Salmonella* contamination prevalence in food products (*F_PREV*) were available.**

We observed a positive association between the prevalence of *Salmonella* in food products and the incidence of human salmonellosis recorded during the study period (β = 0.59; R^2^ = 0.28; *p* < 0.001): the expected percentage change in human illness increased by 0.5% for each one-unit increase in the prevalence of *Salmonella* in food products (Additional file 2, Figure S4).

**Figure 4:**
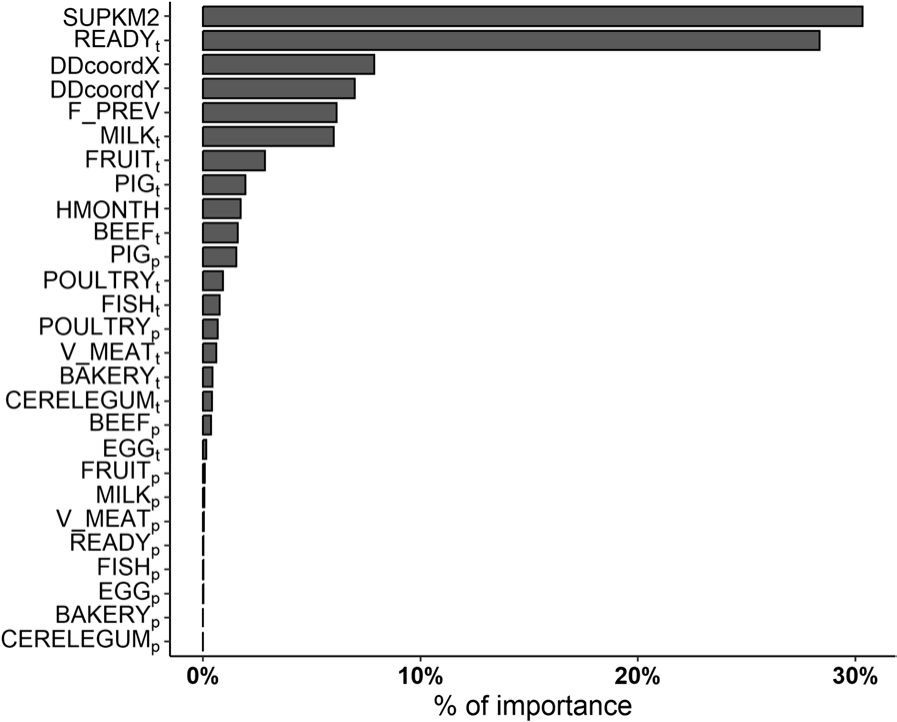
**Feature importance displayed by the random forest (*RF*) algorithm in scenario 1, expressed as the total percentage of variance reduction that results from predictor splits.**

The application of the different ML algorithms to the initial dataset (*n* = 220 records) yielded notable differences in model performance. Table 4 summarises the optimal tuning parameters, the fit and the MAPE results obtained by each algorithm in different scenarios on the test set. *GB* and *RF* generally outperformed the *TR* algorithm; however, all models achieved only low-to-moderate fit levels. No improvements in the performances of the *RF* and *GB* algorithms were observed when they were applied to scenarios with extended time lags; all of these algorithms achieved the highest level of fit in scenario 1. Furthermore, the MAPE for all the models did not decrease but instead increased, especially in scenario 2. We recorded the lowest MAPEs with the highest model fit in scenario 1 (Table 4), indicating that this was the most suitable time lag for prediction with minimal error. Based on this latter result, we fitted the three models with the 2019 integrated dataset containing the time slots and municipalities for which the *H_INC* and *F_PREV* data were complete. This highlighted the differences observed earlier between the three algorithms (Figure 3). We obtained a comparatively low average *H_INC* compared to the total average *H_INC* observed in the study municipalities (*n* = 39; 3.09 per 10 000 person-years). The *TR* algorithm yielded an average incidence rate of 2.07 per 10 000 person-years, and the incidence rates calculated using *RF* and *GB* were 1.85/10 000 and 2.16/10 000, respectively. Among the 27 features included in the models, the relevance of spatial data and the testing effort performed in particular food matrices were prominent (Figure 4), resulting in a 90.5% reduction in the variance of *Salmonella* incidence estimates. The sampling/testing effort employed in ready-to-eat foods (*READY*_*t*_), milk (*MILK*_*t*_), fruit and vegetables (*FRUITt*) and pig meat (*PIG*_*t*_) provided the greatest contribution to the models’ prediction ability. By contrast, the contribution of positive outcomes ascertained for each food category was generally low (2.9%), with the number of positive pig meat samples (*PIG*_*p*_) obtaining the highest level of importance (1.5%; Figure 4).

**Table 4:** **Optimal tuning parameters obtained after conducting five-fold cross-validation for tree regression (*TR*), random forest (*RF*) and gradient boosting (*GB***) **algorithms**

ML algorithm	Scenario	Optimal tuning parameters				Log-scale	
		Tree depth	No. splitting features	N. of trees	Learning rate	Fit	MAPE (%)
*TR*	1	2				0.42	8.8
	2	2				0.18	8.6
	3	1				0.12	9.1
*RF*	1	20	20	150		0.55	7.5
	2	5	4	50		0.32	8.3
	3	5	8	50		0.31	5.8
*GB*	1	3		50	0.1	0.55	7.5
	2	1		50	0.1	0.35	8.3
	3	1		50	0.1	0.27	6.3

The dataset for the years 2015–2018 (*n* = 220 observations) was used, including municipalities with complete information on *H_INC* and *F_PREV*

**Figure 5:**
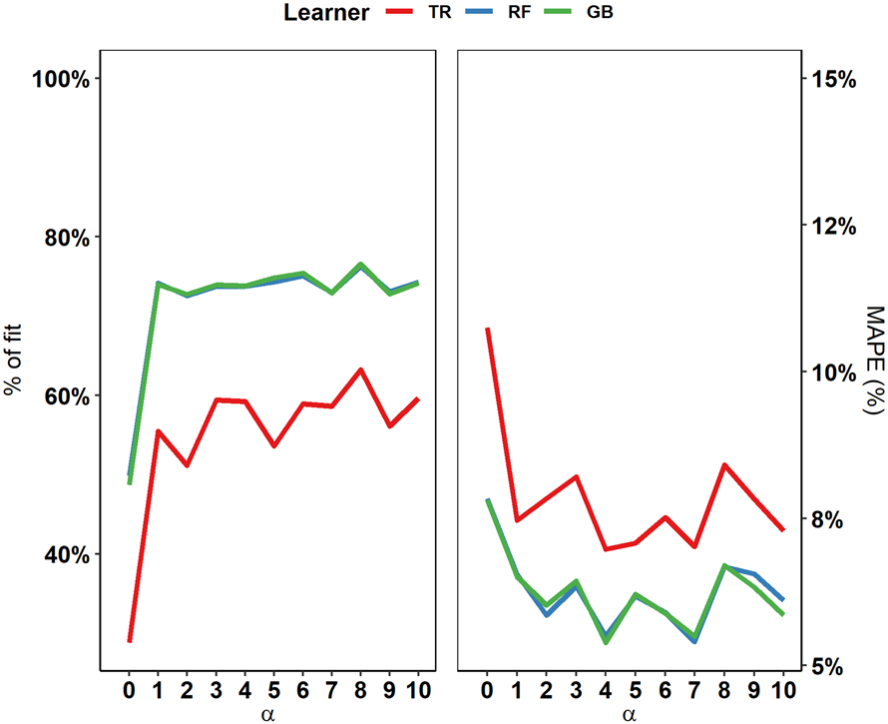
**Calibration of the tree regression (*TR*), random forest (*RF*) and gradient boosting (*GB*) algorithms in scenario 1 at different levels of simulated sampling/testing effort (α).**

By simulating greater data availability regarding *Salmonella* prevalence in food products, we observed that the prediction models displayed better performance and reduced the percentage error. Even minor increases in the simulated sampling/testing effort ($\alpha )$ sufficed to maximise the models’ performance with increased data availability. Nevertheless, no further improvement in model fit occurred with gradual increases in $\alpha$ levels (Figure 5). *RF* and *GB* were verified as the best performing algorithms, achieving comparable levels of fit and attaining optimal performance at an $\alpha$ level = 4 (R^2^ ≈ 0.74; Additional file 2, Sect. 4, Table S3), with MAPE values of 5.50% and 5.39%, respectively. By contrast, *TR* seemed to require more food sampling ($\alpha$ level = 10) to achieve optimal performance (R^2^ ≈ 0.60; Additional file 2, Sect. 4, Table S3) with the lowest error (MAPE = 7.29%).

**Figure 6:**
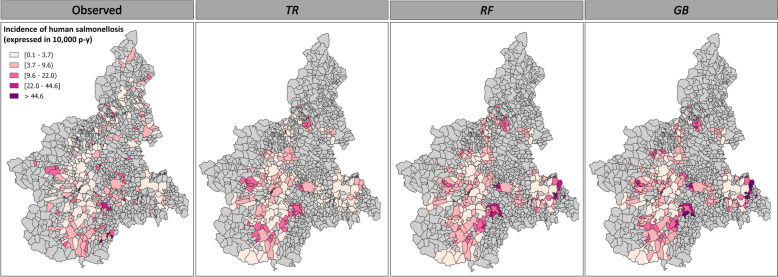
**Comparison of the observed and predicted incidence rates of salmonellosis in humans using tree regression (*TR*), random forest (*RF*) and gradient boosting (*GB*) algorithms with food safety surveillance data from 2019.**

In 2019, health services recorded an average *H_INC* of 7.03 per 10 000 person-years, encompassing 213 out of 1181 municipalities in the Piedmont region. Food safety surveillance covered 337 municipalities and revealed a total food contamination prevalence of 4.1% (95% CI  3.6–4.7) involving 48 municipalities (Additional file 3). Based on the food-recorded data, our models predicted infection rates similar to those observed by the health services, especially when using *RF* (8.08/10 000) and *GB* (10. 4/10 000). However, we obtained lower incidence rates with *TR,* with an estimated average incidence rate of 4.99/10 000. The fit and MAPE of the algorithms for the predicted incidence are illustrated in Table 5. In our dataset, human cases of salmonellosis were officially reported in 213 of a total of 1181 municipalities. As described above, our models were used to predict the occurrence status (in terms of the presence or absence of at least one case) of each municipality. When the disease was reported by the health services, our models showed a sensitivity of 46.5% (99/213). Of the 968 municipalities where no human cases were recorded, the absence of the disease was correctly predicted in 760, yielding a specificity of 78.5% (Figure 6).

**Table 5:** **Performance of the ML algorithms for predictions in 2019**

ML algorithm	Optimal tuning parameters				Log-scale		Natural scale	
	Tree depth	No. splitting features	N. of trees	Learning rate	Fit	MAPE (%)	Fit	MAPE (%)
*TR*	2				0.9998	8.21	0.9840	66.3
*RF*	20	20	150		0.9998	7.37	0.9872	59.9
*GB*	3		50	0.1	0.9999	7.07	0.9887	64.4

## Discussion

By fitting tree-based ML algorithms to real-world data produced by regional food safety surveillance activity, we successfully forecasted spatiotemporal patterns of emerging *Salmonella* infections in the local population of the Piedmont region of northwestern Italy. This novel approach highlights the essential role of veterinarians in the animal-based food supply chain and emphasises the importance of interdisciplinary collaboration in protecting public health [36]. In addition, the method provides a consistent approach that can be implemented in food surveillance databases for hazards beyond *Salmonella* spp.

Despite ongoing efforts to reduce foodborne salmonellosis, its complexity derives from the various pathways that lead to foodborne illnesses and the different food products that can be involved in human outbreaks [37, 38]. ML techniques can support prevention efforts against salmonellosis because these algorithms can detect complex patterns that can be overlooked by conventional methods, especially when dealing with intricate interactions and patterns [39]. Among the tree-based algorithms used, *random forest (RF)* and *gradient boosting (GB)* algorithms exhibited comparable abilities to predict human salmonellosis. Both algorithms outperformed *tree regression (TR)* algorithms in terms of accuracy and reduced uncertainty. *TR* algorithms are known to yield highly unstable predictions, as they are prone to overfitting and show high variance errors [40]. However, the incorporation of bootstrap aggregation techniques into *TR* (resulting in *RF* and *GB* algorithms) enhanced its predictive power and generalisation capabilities [41, 42].

Although the sensitivity levels were quite low and there were quite a few instances where our models inaccurately failed to predict the occurrence of at least one case at the municipality level, both the *RF* and *GB* algorithms were able to identify significant occurrences of actual human salmonellosis. Although the predictions of these ML algorithms closely resembled the infection rates reported by regional clinical laboratories and public health authorities, there were notable discrepancies in the distribution of the disease compared to the observed data. Several reasons may explain these discrepancies. Disease dynamics play a significant role, as cases may arise in municipalities escaping detection by the healthcare system, or the origin of contaminated food may be traced to a different region [43]. Additionally, the high rates of false-negative results recorded may be partly due to constraints imposed by the quality and quantity of collected data available for developing the models [44]. *Salmonella* outbreaks primarily occur in domestic settings. However, disease cases are usually reported based on the municipality of the individual’s official residence. This practice may not accurately reflect the actual home location of the patients, potentially leading to some geographical misclassification of the disease distribution. Such imprecision could impede our efforts to improve the accuracy of our predictions and have a direct impact on the development of health system policies and their subsequent evaluation [45].

Despite these challenges, it is important to acknowledge that our analysis assumes a solid and efficient health care system; however, limitations of the surveillance strategies employed for humans and food products, administrative challenges within the health system, and intrinsic factors related to the natural progression of the illness may also account for the obtained results. Human salmonellosis surveillance operates under a passive monitoring approach, which inherently limits its ability to effectively identify disease cases. This strategy heavily relies on interactions with the health care system to detect cases and is thus subject to such interactions. However, various factors, including an individual’s attitude towards seeking medical care, the prevalence of subclinical or self-limiting infections, and challenges related to diagnosing, communicating and investigating cases within the healthcare system, can undermine the efficiency of case detection [46–49]. Although salmonellosis infections typically resolve spontaneously, certain vulnerable individuals, such as infants, elderly individuals, and immunocompromised individuals, may develop severe forms of the disease that require medical intervention [50, 51]. As a result, only a fraction of illness events within the population are detected, reported and communicated to health authorities [52]. In Greece, it was estimated that only 47.7% of human salmonellosis cases are officially reported, highlighting notable regional differences in disease reporting practices [53]. We noted a comparable situation when incorporating data from health authorities with records from regional laboratories. Our study revealed that although approximately 60% of the cases were officially reported, a significant number of cases were recognised by health services but not conveyed to health authorities. This difference exemplifies the difficulty of achieving extensive and precise data integration across the healthcare system and adequately capturing and measuring the real burden of *Salmonella* infections in the population [54, 55].

On the other hand, food safety surveillance is built upon standardised active monitoring aimed at the timely detection and resolution of potential foodborne hazards [56]. However, the main challenge to this approach has centred on sampling considerations. As microbial contaminants can occur at multiple stages of the food supply chain, the effectiveness of the active surveillance system depends on the accuracy of the sampling process [57, 58]. Therefore, any gaps in active monitoring activities or reduced sampling efforts for food products in certain areas could lead to oversights. This could cause our ML models to miss certain clusters of human salmonellosis (as we noticed in the northeastern and southeastern parts of the Piedmont region). These findings highlight the crucial role of sampling decisions in achieving successful results and emphasise the need to increase efforts within surveillance systems to reduce the risk of contaminated food reaching consumers and to protect public health. There are many types of food in which *Salmonella* spp. are actively searched for, and the accuracy of this search significantly influences the probability of detection. The total number of tests performed on ready-to-eat foods, milk and milk products, fruits and vegetables, and pig meat and its byproducts were among the most important features for predicting human salmonellosis. These findings are consistent with recent European-level zoonotic surveillance data, which highlight mixed foods and pig meat as the primary food categories frequently implicated in human outbreaks [2]. In our study, a prevalence of 1.7% was found in pig meat and its byproducts, indicating a higher level of *Salmonella* contamination compared to the average prevalence observed at both the Italian and European levels. These contrasting results may rely on the broad food categorisation used, as we did not differentiate between different meat products such as carcasses, fresh meat, or minced meat. Consequently, the higher prevalence observed may be due to the more frequent occurrence of the pathogen in pig meat products other than carcasses. In fact, *Salmonella* contamination is most common in non-ready-to-eat foods derived from poultry and pig meat [2]. Nonetheless, the impact of the presence of *Salmonella* in food samples on the prediction of human cases appears to be rather limited, and it is important to be cautious in our interpretation. We recognise that the inclusion of this information contributed to our ability to predict the geographical distribution of documented human outbreaks and the observed incidence rates. However, it is important to recognise that the performance of our models may be influenced by a complex interplay of factors, and the relative importance of certain variables may vary [59].

Data integration is a crucial aspect of gaining insights from real-time data [60]. Surveillance platforms are a reliable source of information on confirmed cases of disease and/or infection compared to other data sources. However, merging data from various sources can be challenging [61]. In our case, the integration of human and food databases was successful due to their similar structure and common fields that facilitated data merging. The challenge at hand was to obtain sufficient data for estimating ML model parameters. It is well known that the size of the dataset used for ML techniques has a significant impact on the precision and accuracy of the predicted outcomes [62, 63]. The first training dataset used in this study contained a limited number of records to ensure data completeness, resulting in models with moderate-to-low performance and low prediction accuracy. To evaluate the models’ effectiveness in predicting disease cases, we simulated increased availability of complete data. The resulting increase in prediction accuracy confirms the models’ suitability for our stated objective. Our implementation of ML techniques underscores their potential to enhance the efficiency of health services [10]. Although our predictive models do not have optimal sensitivity and specificity, the usefulness of these techniques is significant in regard to addressing evolving diseases and changing transmission patterns [64]. ML models can learn and adapt from new data continuously, enhancing their overall usefulness by refining their predictions as new data surfaces [65]. These methods can identify anomalous shifts in data that may indicate an emerging outbreak. Although these models may overlook specific cases, the overall detection of these shifts can help authorities take proactive measures to prevent larger outbreaks [66]. Moreover, the timely identification of potential high-risk outbreak areas and particular food categories that significantly contribute to disease transmission can offer guidance for targeted interventions. This enables health services to focus their efforts on the areas in greatest need, optimising resource allocation in a more responsive and data-driven manner and mitigating the impact of disease spread.

Our findings highlight the significance of interdisciplinary collaboration, reliable data integration, and the utilisation of ML techniques to enhance preparedness to effectively manage risks from foodborne salmonellosis. We have gained valuable insights into the potential of food safety surveillance data in predicting foodborne *Salmonella* outbreaks. Additionally, challenging issues have been identified within healthcare services regarding data transmission and integration, emphasising the complexity of managing epidemiological data. The use of ML algorithms, particularly *random forest* (*RF*) and* gradient boosting *(*GB*), on our dataset has shown considerable success in predicting cases of human salmonellosis. Despite some inherent shortcomings, such as limited sensitivity and specificity, these ML algorithms nevertheless represent valuable operational tools. As such, they hold great promise as a warning resource for public health interventions, thereby facilitating a proactive response. The methodology outlined here offers potential for adaptation to other contexts and communicable diseases. However, any extension of this method should be undertaken carefully, taking into account the specific characteristics and challenges of particular epidemiological scenarios. This approach, if implemented with care and consideration of local epidemiological circumstances, can provide insightful guidance and support in protecting public health.

### Supplementary Information

**Additional file 1. Management and processing of food safety surveillance data. **Flowchart illustrating the retrieval and processing of food safety surveillance data from the SIGLA database, the electronic system of the *Istituto Zooprofilattico Sperimentale del Piemonte*,* Ligura e Valle d’Aosta*.

**Additional file 2. Codes used to perform the analyses presented in the current manuscript and their outputs.**

**Additional file 3. Geographical distribution of food surveillance activity in the Piedmont region in 2019.** Figure illustrating municipalities that were subjected to food surveillance in 2019.

**Additional file 4. Data supporting the results and conclusions of the present manuscript.** Details on the datasets provided are explained in Additional file 2.

## Data Availability

The raw data were generated by the *Servizio di riferimento Regionale di Epidemiologia per la sorveglianza, la prevenzione e il controllo delle Malattie Infettive* (*SeREMI*) and *Istituto Zooprofilattico Sperimentale del Piemonte, Liguria e Valle d’Aosta* (*IZSPLVA*). Access to these data is limited due to licensing agreements for the current study, and these data are not publicly available. The derived data and code scripts on which the conclusions of this study are based are openly available in the Additional files.
