# Supplementary figures and images for: Machine learning approach as an early warning system to prevent foodborne Salmonella outbreaks in northwestern Italy
Source: Vet Res. 2024 Jun 5;55:72. doi: 10.1186/s13567-024-01323-9 (PMC11154984; doi:10.1186/s13567-024-01323-9)

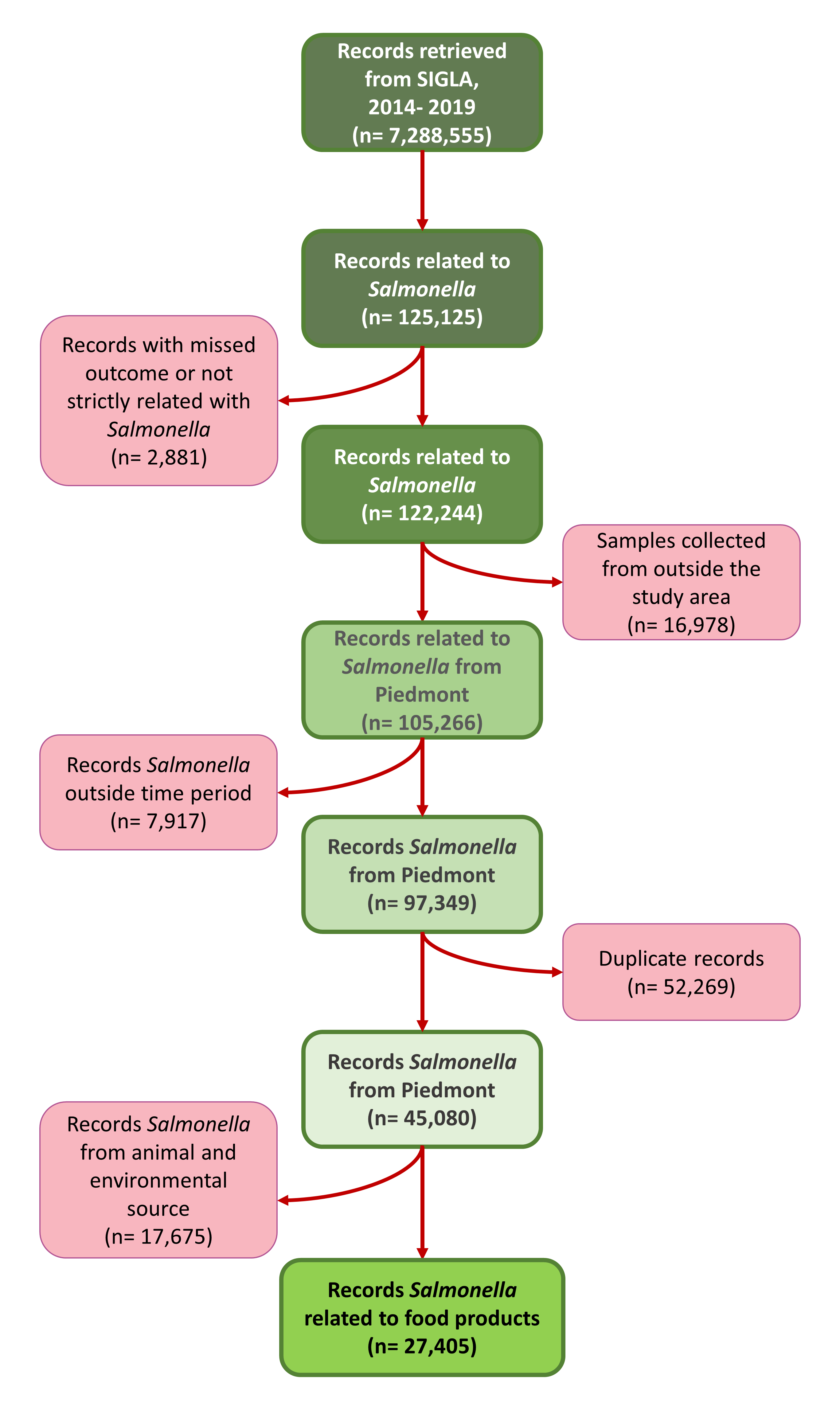

Supplement: Supplementary file 1 — Additional file 1. Management and processing of food safety surveillance data. Flowchart illustrating the retrieval and processing of food safety surveillance data from the SIGLA database, the electronic system of the Istituto Zooprofilattico Sperimentale del Piemonte, Ligura e Valle d’Aosta. [file 13567_2024_1323_MOESM1_ESM.tif]

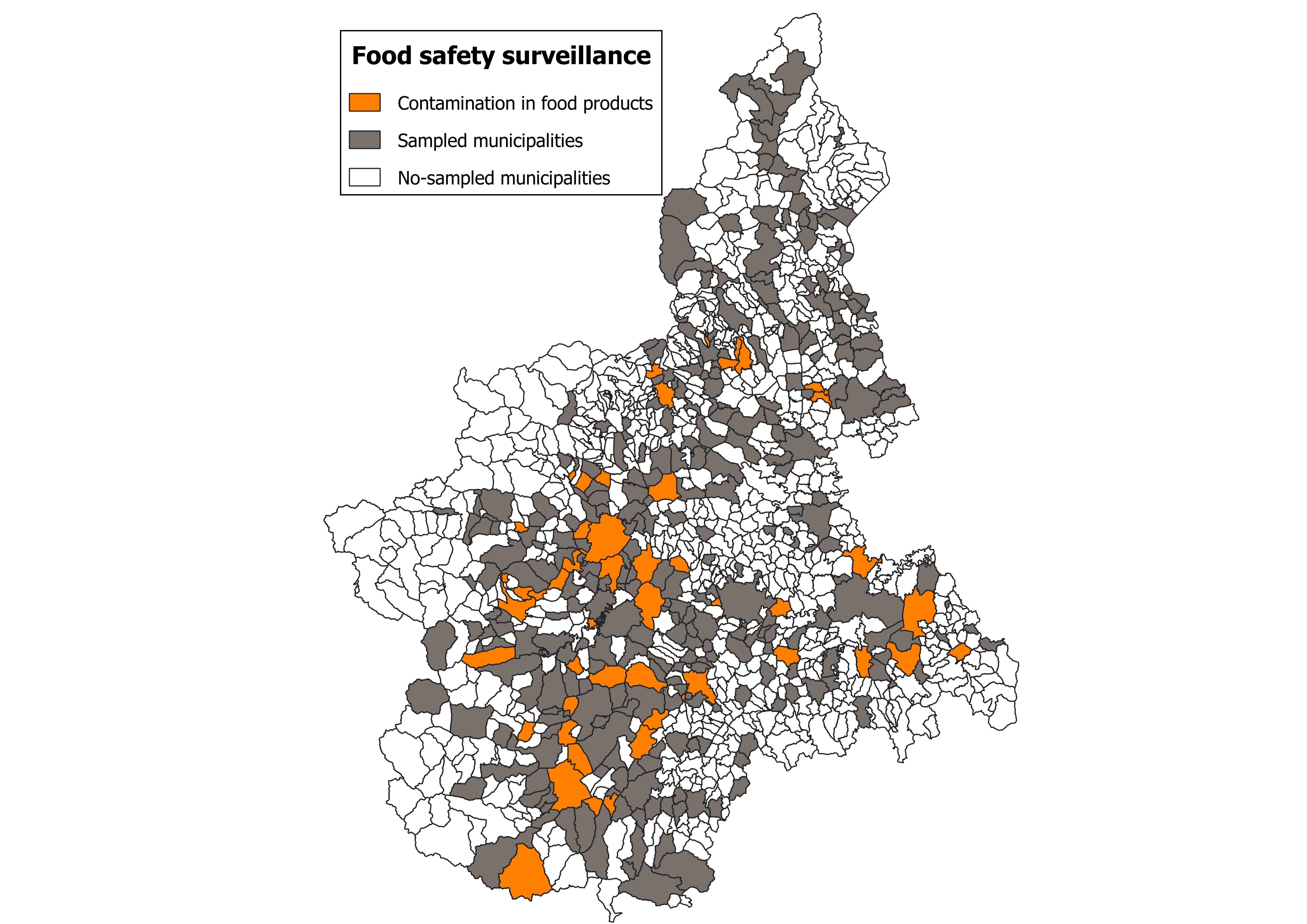

Supplement: Supplementary file 3 — Additional file 3. Geographical distribution of food surveillance activity in the Piedmont region in 2019. Figure illustrating municipalities that were subjected to food surveillance in 2019. [file 13567_2024_1323_MOESM3_ESM.tiff]
