# Supplementary material for: Machine learning approach as an early warning system to prevent foodborne Salmonella outbreaks in northwestern Italy
Source: Vet Res. 2024 Jun 5;55:72. doi: 10.1186/s13567-024-01323-9 (PMC11154984; doi:10.1186/s13567-024-01323-9)
Supplement: Supplementary file 2 — Additional file 2. Codes used to perform the analyses presented in the current manuscript and their outputs. [file 13567_2024_1323_MOESM2_ESM.docx]

Additional file 2: Machine learning approach as an early warning system to prevent foodborne *Salmonella* outbreaks in northwestern Italy

**Introduction**

This supplementary report describes the supervised machine learning (ML) approach developed to estimate the human incidence of foodborne *Salmonella* infections in northwestern Italy in 2019 based on corresponding food safety surveillance data. This report contains both the required code and accompanying data files to repeat the analyses in STATA 17 using open-source Python Distribution Anaconda (Python 3.9).

The analyses required the r_ml_stata_cv command. It is assumed throughout this document that Python software has been installed in its default location and that all model and data files reside in the same working directory. If this is not the case, then adjustments to the code should be made to ensure correct linkage to the necessary files.

The analysis proceeds as follows:

- Initially, we evaluate the relationship between the incidence of salmonellosis in the resident population and food contamination by *Salmonella* spp.
- We evaluate the three predefined temporal scenarios and determine their suitability for predicting human infections.
- Subsequently, we estimate the models’ parameters and attempt to predict the incidence of human infections. We use the epidemiological units provided with available information on both human infections and food contamination, considering the optimal temporal scenario. Predictions are compared with the actual incidence recorded by the health system.
- We assess the performance of the selected ML algorithms using Laplace smoothing, which simulates increased availability of food prevalence data for each epidemiological unit.
- Finally, we predict the incidence of human infections by exclusively using unlabelled data from food safety surveillance in 2019 and considering the most suitable scenario.

**Description of the dataset variables**

The datasets provided (“scen1.csv”, “scen2.csv”, and “scen3.csv”) contain essential data on 34 necessary variables for performing the assessments and analyses described in this document. The description of each variable is given below.

| **Variable name** |  | **Variable label** |
| --- | --- | --- |
|  |  |  |
| *municipality_code* |  | ID code of the municipality |
| *municipality* |  | Municipality name |
| *province* |  | Province acronym |
| *ddcoordx* |  | Longitude of centroid of the municipality (expressed in decimal degrees) |
| *ddcoordy* |  | Latitude of centroid of the municipality (expressed in decimal degrees) |
| *h_year* |  | Year during which human cases occur |
| *h_month* |  | Month during which human cases occur |
| *pop* |  | Average population of the municipality |
| *h_inc* |  | Sex- and age-standardised incidence of foodborne salmonellosis in the specific municipality’s population |
| *km2_sup_tot* |  | Total surface of the exposure area expressed in km^2^ |
| *tot_food_case* |  | Number of contaminated food matrices detected in the exposure area |
| *tot_lab_analysis* |  | Total number of lab analyses conducted in food matrices from the exposure area |
| *egg_t* |  | Total number of lab analyses performed on egg matrices |
| *egg_p* |  | Number of contaminated egg matrices |
| *milk_t* |  | Total number of lab analyses performed on milk matrices |
| *milk_p* |  | Number of contaminated milk matrices |
| *cerelegum_t* |  | Total number of lab analyses performed on cereal-based products and legumes |
| *cerelegum_p* |  | Number of contaminated cereal-based and legumes matrices |
| *fruit_t* |  | Total number of lab analyses performed on fruit and vegetable matrices |
| *fruit_p* |  | Number of contaminated fruit and vegetable matrices |
| *bakery_t* |  | Total number of lab analyses performed on bakery matrices |
| *bakery_p* |  | Number of contaminated bakery matrices |
| *fish_t* |  | Total number of lab analyses performed on fish matrices |
| *fish_p* |  | Number of contaminated fish matrices |
| *poultry_t* |  | Total number of lab analyses performed on poultry meat matrices |
| *poultry_p* |  | Number of contaminated poultry meat matrices |
| *beef_t* |  | Total number of lab analyses performed on beef meat matrices |
| *beef_p* |  | Number of contaminated beef meat matrices |
| *pig_t* |  | Total number of lab analyses performed on pig meat matrices |
| *pig_p* |  | Number of contaminated pig meat matrices |
| *v_meat_t* |  | Total number of lab analyses performed on various meat matrices |
| *v_meat_p* |  | Number of contaminated various meat matrices |
| *ready_t* |  | Total number of lab analyses performed on ready-to-eat products |
| *ready_p* |  | Number of contaminated ready-to-eat matrices |
|  |  |  |

Data on *Salmonella* contamination in food products and the incidence of foodborne *Salmonella* infections for the year 2019 are also provided in food_data_19_scen1.csv and h_inc_2019.csv, respectively.

**Analysis**

1. **Assessing the relationship between the incidence of salmonellosis in humans and the prevalence of food product contamination in the Piedmont region from 2015 to 2018.**

The data are provided as a Supplementary Data File (“scen1.csv”, “scen2.csv”, and “scen3.csv”) online. We imported the data into STATA, calculated the prevalence of food contamination from 2015–2018 and summarised the data based on the number of records for which information on human incidence and food prevalence was available. Here, we provide the results obtained for data scenario 1.

summarize f_prev, d

** Set the directory:*

cd "~ Codes and datasets"

** Load SCENARIO 1 dataset:*

import delimited "C:\ ~Codes and datasets\scen1.csv"

** Delete records from the year 2019:*

drop if h_year == 2019

** Calculate the contamination prevalence in food products:*

gen f_prev = (tot_food_case/ tot_lab_analysis)

** Check missing values in human incidence* (h_inc) *and food contamination prevalence* (prev):

mdesc h_inc f_prev

From these outputs, we note that the calculation of food contamination prevalence (f_prev) has failed for 295 records, as no laboratory analyses on food matrices have been performed. The summary of f_prev values highlights that for most of the epidemiological units (n= 862), the value of f_prev is equal to zero. Therefore, the number of records for which we have complete information on both h_inc and f_prev is 220.

Now, we evaluate how h_inc and f_prev are distributed and their relationship:

** Display the distribution of h_inc and prev values:*

histogram h_inc, frequency fcolor(gs11) lcolor(black) ylabel(, labcolor(black) tlcolor(black) nogrid glcolor()) xlabel(, labcolor(black) tlcolor(black) nogrid) scheme(s2mono)

histogram f_prev, frequency fcolor(gs11) lcolor(black) ylabel(, labcolor(black) tlcolor(black) nogrid glcolor()) xlabel(, labcolor(black) tlcolor(black) nogrid) scheme(s2mono)

*Figure S1. Distribution of the calculated* h_inc and prev *values*

It is apparent from the output that both h_inc and f_prev have a right-skewed distribution with long tails. It could be challenging to identify whether there is some type of relation between the two variables. For this reason, we log-transform both the h_inc and f_prev variables to assess how they behave.

** Log-transformation of* h_inc *and* prev:

gen l_inc=log(h_inc)

gen l_prev=log(f_prev)

** Display the distribution of* l_inc *and* l_prev *values:*

histogram l_inc, frequency fcolor(gs11) lcolor(black) ylabel(, labcolor(black) tlcolor(black) nogrid glcolor()) xlabel(, labcolor(black) tlcolor(black) nogrid) scheme(s2mono)

histogram l_prev, frequency fcolor(gs11) lcolor(black) ylabel(, labcolor(black) tlcolor(black) nogrid glcolor()) xlabel(, labcolor(black) tlcolor(black) nogrid) scheme(s2mono)

*Figure S2. Distribution of* l_inc *and* l_prev *values*

In logarithmic form, there appears to be a linear relationship between l_inc and l_prev, as illustrated by the scatter plot. To confirm this association, we conduct a log-log linear regression as follows:

Source | SS df MS Number of obs = 220

-------------+---------------------------------- F(1, 218) = 85.56

Model | 110.267179 1 110.267179 Prob > F = 0.0000

Residual | 280.966088 218 1.28883527 R-squared = 0.2818

-------------+---------------------------------- Adj R-squared = 0.2786

Total | 391.233267 219 1.78645327 Root MSE = 1.1353

------------------------------------------------------------------------------

l_inc | Coefficient Std. err. t P>|t| [95% conf. interval]

-------------+----------------------------------------------------------------

l_prev | .5927426 .0640828 9.25 0.000 .4664414 .7190438

_cons | -8.343943 .1637281 -50.96 0.000 -8.666635 -8.02125

------------------------------------------------------------------------------

regress l_inc l_prev

*Figure S3. Relationship between* l_inc *and* l_prev

** Evaluate the relationship between* l_inc *and* l_prev*:*

twoway (scatter l_inc l_prev, mcolor(black) msize(small) msymbol(circle)), scheme(s2mono)

The output of our model indicates a slight linear relationship between l_inc and l_prev (β = 0.59), as shown in the graph below:

twoway (scatter l_inc l_prev, mcolor(black) msize(small) msymbol(circle)) (lfitci l_inc l_prev, ciplot(rline)), scheme(s2mono)

*Figure S4. Linear fit prediction plot for* l_inc *on* l_prev *in the Piedmont region from 2015 to 2018, with 95% CIs of the prediction*

1. **The optimal temporal scenario for predicting human incidence was assessed by using food prevalence recorded from 2014 to 2018 and human incidence recorded from 2015 to 2018.**

As detailed in the manuscript, we devised three temporal scenarios with different time lags to consider the time elapsed between pathogen exposure and identification as a case by health services. The time lags defined for each scenario were two months for scenario 1, four months for scenario 2 and seven months for scenario 3.

After loading the corresponding datasets, we randomly divided the data into training and test sets at a 7:3 ratio. Subsequently, we establish the dependent variable and the potential predictor variables and fit the selected ML algorithms with the uploaded data.

** Load SCENARIO 1 dataset:*

clear

import delimited "C:\ ~Codes and datasets\scen1.csv"

** Delete records from the year 2019:*

drop if h_year == 2019

** Calculate the contamination prevalence in food products:*

gen f_prev = (tot_food_case/ tot_lab_analysis)

** Log-transformation of the calculated prevalence in food products and Salmonella incidence in humans:*

gen l_inc=log(h_inc)

gen l_prev=log(f_prev)

** Delete non informative records (i.e., where no values for* l_prev *are provided):*

drop if l_prev ==.

** Form the train and test datasets:*

get_train_test , dataname("scen1") split(0.70 0.30) split_var(svar) rseed(101)

** Form the target (the dependent variable) and the features (the potential predictors):*

global y "l_inc"

global X "ddcoordx ddcoordy h_month km2_sup_tot l_prev egg_t egg_p milk_t milk_p cerelegum_t cerelegum_p fruit_t fruit_p bakery_t bakery_p fish_t fish_p poultry_t poultry_p beef_t beef_p pig_t pig_p v_meat_t v_meat_p ready_t ready_p"

** Run* ***tree regression*** ***(TR)*** *with cross-validated tree depth*

cap rm CV.dta

use scen1_train , clear

r_ml_stata_cv $y $X , mlmodel("tree") data_test("scen1_test") prediction("pred") tree_depth(1 2 3 4 5 6 7 8 9 10 11 12 13 14 15 16 17 18 19 20 21 22 23 24 25) cross_validation("CV") n_folds(5) seed(10)

** Run* ***random forest regression (RF)*** *with cross-validation*

cap rm CV.dta

use scen1_train , clear

r_ml_stata_cv $y $X , mlmodel("randomforest") data_test("scen1_test") tree_depth(5 10 15 20 25) n_estimators(50 150 250) max_features(1 2 3 4 5 6 7 8 9 10 11 12 13 14 15 16 17 18 19 20 21 22 23 24 25 26 27) prediction("pred") cross_validation("CV") n_folds(5) seed(10)

** Run* ***gradient boosting regression (GB)*** *with cross-validation*

cap rm CV.dta

use scen1_train , clear

r_ml_stata_cv $y $X , mlmodel("boost") data_test("scen1_test") tree_depth(1 2 3 4 5 6 7 8 9 10 11 12 13 14 15 16 17 18 19 20 21 22 23 24 25) n_estimators(50 150 250) learning_rate(0.1 0.3) prediction("pred") cross_validation("CV") n_folds(5) seed(10)

--------------------------------------------------------------------------------

Learner: Tree regression

Dataset information

Target variable = "l_inc" Number of features = 27

N. of training units = 154 N. of testing units = 66

N. of used training units = 154 N. of used testing units = 66

--------------------------------------------------------------------------------

Cross-validation results

Accuracy measure = explained variance Number of folds = 5

Best grid index = 1 Optimal tree depth = 2

Training accuracy = .64861327 Testing accuracy = .42187371

Std. err. test accuracy = .26268025

--------------------------------------------------------------------------------

Validation results

MSE = mean squared error MAPE = mean absolute percentage error

Training MSE = .61642755 Testing MSE = 1.1538791

Training MAPE % = 6.3899405 Testing MAPE % = 8.8473622

--------------------------------------------------------------------------------

**Learner: Random Forest regression**

Dataset information

Target variable = "l_inc" Number of features = 27

N. of training units = 154 N. of testing units = 66

N. of used training units = 154 N. of used testing units = 66

--------------------------------------------------------------------------------

Cross-validation results

Accuracy measure = rate correct matches Number of folds = 5

Best grid index = 341 Optimal tree depth = 20

Optimal n. of splitting features = 20 Optimal n. of trees = 150

Training accuracy = .95798772 Testing accuracy = .54726747

Std. err. test accuracy = .2743081

--------------------------------------------------------------------------------

Validation results

MSE = mean squared error MAPE = mean absolute percentage error

Training MSE = .0729409 Testing MSE = .92469218

Training MAPE % = 2.2201903 Testing MAPE % = 7.5106773

--------------------------------------------------------------------------------

**Learner: Boosting regression**

Dataset information

Target variable = "l_inc" Number of features = 27

N. of training units = 154 N. of testing units = 66

N. of used training units = 154 N. of used testing units = 66

--------------------------------------------------------------------------------

Cross-validation results

Accuracy measure = explained variance Number of folds = 5

Best grid index = 6 Optimal learning rate = .1

Optimal n. of trees = 50 Optimal tree depth = 3

Training accuracy = .95655848 Testing accuracy = .5463506

Std. err. test accuracy = .23574089

--------------------------------------------------------------------------------

Validation results

MSE = mean squared error MAPE = mean absolute percentage error

Training MSE = .1002283 Testing MSE = .91941323

Training MAPE % = 2.7003387 Testing MAPE % = 7.5164213

--------------------------------------------------------------------------------

The output provides details on the performance of the three ML models in the training and test sets:

- The number of records allocated to the training set (*n* = 154) and the test set (*n*= 66).
- The effective number of records that were used to develop the model. In this case, all the records provided were used to generate parameter estimates. This approach is useful for checking whether missing values are present in our datasets.
- The cross-validation results display the optimal parameter estimates and the performances of the models on both the training and test sets. The testing accuracy is expressed as R^2^, and the uncertainty is expressed as the mean average percentage error (MAPE).

The same code rearranged for scenarios 2 and 3 displays the results summarised in the Table below, which corresponds with Table 4 of the manuscript.

*Table S1. Optimal tuning parameters obtained after conducting 5-fold cross-validation for tree regression (TR), random forest (RF) and gradient boosting (GB) algorithms. The dataset for the years 2015-2018 (n= 220 observations) was used, including municipalities with complete information on* h_inc *and* f_prev*.*

| **ML algorithms** | **Scenario** | **Optimal tuning parameters** | | | | **Real prevalence** | |
| --- | --- | --- | --- | --- | --- | --- | --- |
|  |  | **Tree depth** | **No. splitting features** | **N. of trees** | **Learning rate** | **Fit** | **MAPE (%)** |
|  |  |  |  |  |  |  |  |
| ***TR*** | 1 | 2 |  |  |  | 0.42 | 8.8 |
|  | 2 | 2 |  |  |  | 0.18 | 8.6 |
|  | 3 | 1 |  |  |  | -0.12 | 9.1 |
|  |  |  |  |  |  |  |  |
|  |  |  |  |  |  |  |  |
| ***RF*** | 1 | 20 | 20 | 150 |  | 0.55 | 7.5 |
|  | 2 | 5 | 4 | 50 |  | 0.32 | 8.3 |
|  | 3 | 5 | 8 | 50 |  | 0.31 | 5.8 |
|  |  |  |  |  |  |  |  |
|  |  |  |  |  |  |  |  |
| ***GB*** | 1 | 3 |  | 50 | 0.1 | 0.55 | 7.5 |
|  | 2 | 1 |  | 50 | 0.1 | 0.35 | 8.3 |
|  | 3 | 1 |  | 50 | 0.1 | 0.27 | 6.3 |
|  |  |  |  |  |  |  |  |
|  |  |  |  |  |  |  |  |
|  |  |  |  |  |  |  |  |

Based on the results obtained, we can conclude that Scenario 1 is the best scenario for predicting the incidence of human salmonellosis, as we obtained the highest model accuracy with minimal uncertainty.

1. **Predicting the human incidence of salmonellosis with 2019 food data considering the optimal temporal scenario and parameters and the epidemiological units for which the data were complete (*n*= 220 observations).**

In this section, we aim to estimate the incidence of human salmonellosis in 2019 using scenario 1 and the data used thus far (*n* = 220) to evaluate the prediction ability of the generated models. Moreover, we assess the relevance of the potential predictors in the model’s performance by displaying the feature importance offered by the RF algorithm. It is essential to install the extension “pylearn” to display the feature importance:

* net install pylearn, from(https://raw.githubusercontent.com/mdroste/stata-pylearn/master/src/) replace

** Load SCENARIO 1 dataset:*

clear

import delimited "C:\ ~Codes and datasets\scen1.csv"

** Calculate the contamination prevalence in food products:*

gen f_prev = (tot_food_case/ tot_lab_analysis)

** Log-transformation of the calculated prevalence in food products and Salmonella incidence in humans:*

gen l_inc=log(h_inc)

gen l_prev=log(f_prev)

** Deleting records for which non information on food prevalence is available:*

gen mis = 1 if l_prev !=.

tab h_year mis, m

drop if mis ==.

** Defining the training, test and unlabelled datasets:*

preserve

keep if h_year == 2019

** Create the label 'sample' of the dataset:*

gen sample = "unlabeled"

** Create the y variable that have to be predicted:*

gen inc =.

save unlabeled_data , replace

restore

drop if h_year == 2019

** Form the train and test datasets:*

get_train_test , dataname("scen1") split(0.70 0.30) split_var(svar) rseed(101)

** Create the label 'sample' and the y variable equal to l_inc that served for the model learning from the training and test datasets:*

sysuse scen1_test, clear

gen sample = "test"

gen inc = l_inc

save scen1_test , replace

sysuse scen1_train, clear

gen sample = "train"

gen inc = l_inc

save scen1_train , replace

**** Run random forest (RF) predicted values:***

cap rm CV.dta

use scen1_train , clear

r_ml_stata_cv $y $X , mlmodel("randomforest") data_test("unlabeled_data") tree_depth(20) n_estimators(150) max_features(20) prediction("pred") cross_validation("CV") n_folds(5) seed(10)

** Save optimal tuning parameters into globals*

global p_depth=e(OPT_MAX_DEPTH)

global p_features=e(OPT_MAX_FEATURES)

global p_estimators=e(OPT_N_ESTIMATORS)

** Compute Feature Importance (using* "pyforest"*)*

pyforest $y $X, type(regress) max_depth($p_depth) n_estimators($p_estimators) max_features($p_features)

matlist e(importance) , rowtitle("Features") title("FEATURE IMPORTANCE")

** Run* ***tree regression (TR)*** ***predicted values:***

cap rm CV.dta

use scen1_train , clear

r_ml_stata_cv $y $X , mlmodel("tree") data_test("unlabeled_data") prediction("pred") tree_depth(2) cross_validation("CV") n_folds(5) seed(10)

** We list the (optimal) predictions over unlabeled data:*

list inc sample pred if inc==.

** Recovery of the incidence values predicted by the model in its anti-logarithmic form.*

** 1) Delete the data belonging to the train_data set:*

drop if sample == "train"

** 2) Generate a new variable with the antilog form of the predicted values:*

gen inc_pred = exp(pred)

** 3) Collapsing the predicted incidence at the municipality level:*

collapse (sum) inc_pred h_inc, by(municipality_code municipality province ddcoordx ddcoordy)

** 4) Expressing the result in 10,000 person-year:*

replace inc_pred =inc_pred*10000

replace h_inc = h_inc*10000

** 5) Display the annual mean incidence rate in real and predicted data:*

list h_inc inc_pred, mean(inc_pred h_inc)

** NOTE: This is the dataset used for mapping the incidence rates predicted by TR algorithm, as illustrated in Figure 3.*

** Form the target (the dependent variable) and the features (the potential predictors):*

global y "inc"

global X "ddcoordx ddcoordy h_month km2_sup_tot l_prev egg_t egg_p milk_t milk_p cerelegum_t cerelegum_p fruit_t fruit_p bakery_t bakery_p fish_t fish_p poultry_t poultry_p beef_t beef_p pig_t pig_p v_meat_t v_meat_p ready_t ready_p"

The matlist function produces a table summarising the importance score for all predictors in the model, which is measured from 0 to 1. As shown in the output and illustrated in Figure 4 of the manuscript, spatial data (ddcoordx, ddcoordy), the surface of the exposure area (km2_sup_tot) together with the prevalence of contamination in food products (l_prev) and the sampling/testing effort undertaken on ready-to-eat products (ready_t), milk (milk_t), fruit and vegetables (fruit_t) and pig meat (pig_t) were found to have the greatest influence on predicting human salmonellosis, leading to a 90.5% reduction in variance.

**** Please note that slight variations in the assigned values for each descriptor may occur in each code run due to the random process; however, this does not alter the resulting feature importance. The command options do not include a* seed() *function.*

Here, we will recover the incidence rates predicted using the *RF* model:

**** Run gradient boosting (GB) predicted values:***

cap rm CV.dta

use scen1_train , clear

r_ml_stata_cv $y $X , mlmodel("boost") data_test("unlabeled_data") tree_depth(3) n_estimators(50) learning_rate(0.1) prediction("pred") cross_validation("CV") n_folds(5) seed(10)

** We list the (optimal) predictions over unlabeled data*

list inc sample pred if inc==.

** Recovery of the value of the incidence predicted by the model in its anti-logarithmic form.*

** 1) Delete the data from the* train_data *dataset:*

drop if sample == "train"

** 2) Generate a new variable with the antilog form of the predicted values:*

gen inc_pred = exp(pred)

** 3) Collapsing the predicted incidence at the municipality level:*

collapse (sum) inc_pred h_inc, by(municipality_code municipality province ddcoordx ddcoordy)

** 4) Expressing the result in 10,000 person-year:*

replace inc_pred =inc_pred*10000

replace h_inc = h_inc*10000

** 5) Display the annual mean incidence rate in real and predicted data:*

list h_inc inc_pred, mean(inc_pred h_inc)

** NOTE: This is the dataset used for mapping the incidence rates predicted by GB algorithm, as illustrated in Figure 3.*

** We list the (optimal) predictions over unlabeled data*

list inc sample pred if inc==.

** Recovery of the value of the incidence predicted by the model in its anti-logarithmic form.*

** 1) Delete the data from the* train_data *dataset:*

drop if sample == "train"

** 2) Generate a new variable with the antilog form of the predicted values:*

gen inc_pred = exp(pred)

** 3) Collapsing the predicted incidence at the municipality level:*

collapse (sum) inc_pred h_inc, by(municipality_code municipality province ddcoordx ddcoordy)

** 4) Expressing the result in 10,000 person-year:*

replace inc_pred =inc_pred*10000

replace h_inc = h_inc*10000

** 5) Display the annual mean incidence rate in real and predicted data:*

list h_inc inc_pred, mean(inc_pred h_inc)

** NOTE: This is the dataset used for mapping the incidence rates predicted by RF algorithm, as illustrated in Figure 3.*

1. **The performances of the tree regression (TR), random forest (RF), and gradient boosting (GB) algorithms in scenario 1 were assessed using simulated data representing different levels of sampling/testing effort (alpha).**

***FOREWORD:*** *The primary aim of this experimental simulation was to evaluate the models' performance with a wider range of food prevalence data, regardless of the food matrices used. Therefore, for simplicity, only the centroid coordinates (*ddcoordx, ddcoordy*), month (*h_month*), exposure area surface (*km2_sup_tot*), and simulated Salmonella prevalence in food products (*l_prev*) were considered.*

In this section, we evaluate how models perform with increased data availability. The entire dataset (scen1.csv; *n* = 1377 epidemiological units) is utilised, including the epidemiological units without recorded prevalence values. As stated in the Materials and methods section of the manuscript, we estimate a hypothetical prevalence of contamination in food products by applying Laplace smoothing. This approach assumes that a greater number of samples, or tests, are needed to detect *Salmonella* spp. in food products. The number of additional samples or tests is set according to five different groups of municipalities based on population size. Table S2 presents the alpha values used in ten different rounds (representing a gradual increase in food sampling/testing in each round) to calculate the ‘corrected’ prevalence ($p_{c}$). The results of the ten rounds are reported in Table S3 and illustrated in Figure 5 of the manuscript.

*Table S2.* Combinations of α values employed to optimise the performance of the ML algorithms in scenario 1.

|  | **SCENARIO 1** | | | | |
| --- | --- | --- | --- | --- | --- |
| **α levels** | **Municipality groups** | | | | |
|  | ***1*** | ***2*** | ***3*** | ***4*** | ***5*** |
| **1** | 1 | 3 | 5 | 7 | 9 |
| **2** | 2 | 6 | 10 | 14 | 18 |
| **3** | 3 | 9 | 15 | 21 | 27 |
| **4** | 4 | 12 | 20 | 28 | 36 |
| **5** | 5 | 15 | 25 | 35 | 45 |
| **6** | 6 | 18 | 30 | 42 | 54 |
| **7** | 7 | 21 | 35 | 49 | 63 |
| **8** | 8 | 24 | 40 | 56 | 72 |
| **9** | 9 | 27 | 45 | 63 | 81 |
| **10** | 10 | 30 | 50 | 70 | 90 |

Note: “α” is defined as the number of additional tests, or samplings, that would have been necessary to detect *Salmonella* in food products. Municipality groups are defined as follows: 1) ≤ 5000 inhabitants; 2) 5001–9999; 3) 10 000–19 999; 4) 20 000–29 999; and 5) ≥ 30 000.

The presented code provides an overview of the steps performed using data of α-level = 1. To evaluate the performance at different α levels, it is necessary to repeat the process by adjusting the code with the values provided for the other α levels. Additionally, it should be noted that the baseline results are achieved by running the code models described in Section 2 (pp. 10-11) after assigning the variables "ddcoordx ddcoordy h_month km2_sup_tot l_prev" to the “global X”.

** Load SCENARIO 1 dataset:*

clear

import delimited "C:\ ~Codes and datasets\scen1.csv"

drop if h_year==2019

sort h_year h_month

gen l_inc=log(h_inc)

**Application of Laplace smothing to calculate the simulated contamination prevalence:*

* *1) Create a new variable with the total analyses carried out for each month every year (valutes reported in Table S2):*

egen total_lab_analysis_month = total(tot_lab_analysis), by(h_year h_month)

** 2) Create a new variable with the total cases of food contamination uncovered for each month every year:*

egen total_food_case_month = total(tot_food_case), by(h_year h_month)

* 3) Create a new variable with the mean prevalence:

gen mu_prev = total_food_case_month/total_lab_analysis_month

* 4) Create a new variable to imputare the hypthetical number of additional analyses/samplings needed to detect food contamination based on the resident population:

gen alfa = .

replace alfa = 1 if pop <=5000

replace alfa = 3 if pop >5000 & pop<10000

replace alfa = 5 if pop >10000 & pop < 20000

replace alfa = 7 if pop >= 20000 & pop < 30000

replace alfa = 9 if pop >=30000

** Calculate the new 'corrected' prevalence (Laplace smoothing formula):*

gen c_prev = ((tot_food_case+(alfa*mu_prev))/(tot_lab_analysis+alfa))

** Log-transformation of the calculated 'corrected' prevalence in food products:*

gen l_c_prev = log(c_prev)

** Form the train and test datasets:*

get_train_test , dataname("scen1") split(0.70 0.30) split_var(svar) rseed(101)

** Form the target (the dependent variable) and the features (the predictors):*

global y "l_inc"

global X "ddcoordx ddcoordy h_month km2_sup_tot l_c_prev"

** Run* ***tree regression*** ***(TR)*** *with cross-validated tree depth*

cap rm CV.dta

use scen1_train , clear

r_ml_stata_cv $y $X , mlmodel("tree") data_test("scen1_test") prediction("pred") tree_depth(1 2 3 4 5 6 7 8 9 10 11 12 13 14 15 16 17 18 19 20 21 22 23 24 25) cross_validation("CV") n_folds(5) seed(10)

** Run* ***random forest regression (RF)*** *with cross-validation*

cap rm CV.dta

use scen1_train , clear

r_ml_stata_cv $y $X , mlmodel("randomforest") data_test("scen1_test") tree_depth(5 10 15 20 25) n_estimators(50 150 250) max_features(1 2 3 4 5) prediction("pred") cross_validation("CV") n_folds(5) seed(10)

** Run* ***gradient boosting regression (GB)*** *with cross-validation*

cap rm CV.dta

use scen1_train , clear

r_ml_stata_cv $y $X , mlmodel("boost") data_test("scen1_test") tree_depth(1 2 3 4 5 6 7 8 9 10 11 12 13 14 15 16 17 18 19 20 21 22 23 24 25) n_estimators(50 150 250) learning_rate(0.1 0.3) prediction("pred") cross_validation("CV") n_folds(5) seed(10)

***Table S3***. Fit and mean absolute percentage error (MAPE) derived from ML learning algorithms in simulated scenario 1, considering different α levels. These levels correspond to hypothetical additional numbers of tests or sampling combinations required for *Salmonella* detection in food products, categorised according to predefined municipality groups.

| $\boldsymbol{\alpha}$ **Level** | ***Tree Regression*** | | |  | ***Random Forest*** | | | | |  | ***Gradient Boosting*** | | | | |
| --- | --- | --- | --- | --- | --- | --- | --- | --- | --- | --- | --- | --- | --- | --- | --- |
|  | **Fit** | **MAPE**  **(%)** | ***od*** |  | **Fit** | **MAPE**  **(%)** | ***od*** | ***omf*** | ***ne*** |  | **Fit** | **MAPE**  **(%)** | ***od*** | ***ne*** | ***lr*** |
|  |  |  |  |  |  |  |  |  |  |  |  |  |  |  |  |
| **Baseline** | 0.28818 | 10.75545 | 7 |  | 0.49876 | 7.84394 | 10 | 5 | 150 |  | 0.48711 | 7.81485 | 2 | 50 | 0.1 |
| **1** | 0.55441 | 7.47132 | 8 |  | 0.74159 | 6.55301 | 20 | 3 | 150 |  | 0.74001 | 6.50691 | 4 | 250 | 0.1 |
| **2** | 0.51168 | 7.84044 | 7 |  | 0.72568 | 5.85534 | 15 | 4 | 150 |  | 0.72763 | 6.02854 | 7 | 50 | 0.1 |
| **3** | 0.59385 | 8.20955 | 8 |  | 0.73724 | 6.34876 | 15 | 3 | 250 |  | 0.73913 | 6.43476 | 6 | 50 | 0.1 |
| 4 | 0.59217 | 6.98094 | 8 |  | **0.73728** | **5.50204** | **25** | **3** | **250** |  | **0.73828** | **5.38781** | **7** | **50** | **0.1** |
| **5** | 0.53647 | 7.08311 | 8 |  | 0.74276 | 6.18187 | 15 | 3 | 150 |  | 0.74811 | 6.20923 | 5 | 150 | 0.1 |
| **6** | 0.58924 | 7.51448 | 8 |  | 0.75057 | 5.90465 | 15 | 4 | 250 |  | 0.75429 | 5.88761 | 5 | 100 | 0.1 |
| **7** | 0.58642 | 7.02868 | 8 |  | 0.73001 | 5.40624 | 25 | 3 | 250 |  | 0.72896 | 5.49529 | 6 | 50 | 0.1 |
| **8** | 0.63209 | 8.41131 | 7 |  | 0.76284 | 6.67946 | 25 | 3 | 150 |  | 0.76527 | 6.70775 | 5 | 250 | 0.1 |
| **9** | 0.56132 | 7.83509 | 11 |  | 0.73091 | 6.55697 | 25 | 4 | 150 |  | 0.72813 | 6.33377 | 5 | 150 | 0.1 |
| **10** | **0.59644** | **7.29126** | **11** |  | 0.74272 | 6.10884 | 20 | 3 | 50 |  | 0.74157 | 5.85831 | 6 | 50 | 0.1 |
|  |  |  |  |  |  |  |  |  |  |  |  |  |  |  |  |

Note: Parameters highlighted in **bold** indicate the highest model performance with minor percentage errors.

*od* = Optimal tree depth

*omf* = Optimal maximum number of features

*ne* = Number of estimators

*lr* = Learning rate

1. **Predicting the incidence of foodborne salmonellosis in humans based on 2019 food data, considering the optimal temporal scenario and parameters.**

In this section, we attempt to predict the incidence of foodborne salmonellosis in the resident population exclusively using data procured from the 2019 food safety surveillance activity. We will implement the models generated in Section 2 and consider the best temporal scenario (scenario 1). The predicted data were then compared with the actual incidence of salmonellosis in the resident population, which is provided in the dataset ‘h_inc_2019.csv’.

** Load SCENARIO 1 dataset and set the training set necessary to fit the model:*

clear

import delimited "C:\ ~Codes and datasets\scen1.csv"

drop if h_year==2019

** Calculate the contamination prevalence in food products:*

gen f_prev = (tot_food_case/ tot_lab_analysis)

** Log-transformation of the calculated prevalence in food products and Salmonella incidence in humans:*

gen l_inc=log(h_inc)

gen l_prev=log(f_prev)

** Deleting records for which non information on food prevalence is available:*

gen mis = 1 if l_prev !=.

tab h_year mis, m

drop if mis ==.

** Form the train and test datasets:*

get_train_test , dataname("scen1") split(0.70 0.30) split_var(svar) rseed(101)

** Set and arrange of the unlabeled dataset:*

** Load the 2019 food safety dataset:*

clear

import delimited "C:\ ~Codes and datasets\food_data_19_scen1.csv"

** Calculate the contamination prevalence in food products:*

gen f_prev = (tot_food_case/ tot_lab_analysis)

** Log-transformation of the calculated prevalence:*

gen l_prev=log(f_prev)

** Drop the records where no data on prevalence is available:*

gen mis = 1 if l_prev !=.

tab h_month mis, m

drop if mis ==.

drop mis

** Generate the dependent variable (incidence in humans) that will be predicted:*

gen l_inc = .

** This corresponds to our unlabeled dataset, which will be used for the predictions:*

save "unlabeled_data", replace

** Form the target (the dependent variable) and the features (the predictors):*

global y "l_inc"

global X "h_month ddcoordx ddcoordy km2_sup_tot l_prev egg_t egg_p milk_t milk_p cerelegum_t cerelegum_p fruit_t fruit_p bakery_t bakery_p fish_t fish_p poultry_t poultry_p beef_t beef_p pig_t pig_p v_meat_t v_meat_p ready_t ready_p"

**** Run random forest (RF) predicted values:***

cap rm CV.dta

use scen1_train , clear

r_ml_stata_cv $y $X , mlmodel("randomforest") data_test("unlabeled_data") tree_depth(20) n_estimators(150) max_features(20) prediction("pred") cross_validation("CV") n_folds(5) seed(10)

ereturn list

** Recovery of the incidence values predicted by the model in its anti-logarithmic form.*

** 1) Delete the data belonging to the train_data set:*

drop if _train_index == "train"

** 2) Generate a new variable with the antilog form of the predicted values:*

gen inc_pred = exp(pred)

** 3) Collapsing the predicted incidence at the municipality level:*

collapse (sum) inc_pred h_inc, by(municipality_code municipality province ddcoordx ddcoordy)

** 4) Expressing the result in 10,000 person-year:*

replace inc_pred =inc_pred*10000

** 5) Display the annual mean incidence rate in the predicted data:*

list inc_pred, mean(inc_pred)

** NOTE: This is the dataset used for mapping the incidence rates predicted by RF algorithm, as illustrated in Figure 6.*

** Run* ***tree regression (TR)*** ***predicted values:***

cap rm CV.dta

use scen1_train , clear

r_ml_stata_cv $y $X , mlmodel("tree") data_test("unlabeled_data") prediction("pred") tree_depth(2) cross_validation("CV") n_folds(5) seed(10)

ereturn list

** Recovery of the incidence values predicted by the model in its anti-logarithmic form.*

** 1) Delete the data belonging to the train_data set:*

drop if _train_index == "train"

** 2) Generate a new variable with the antilog form of the predicted values:*

gen inc_pred = exp(pred)

** 3) Collapsing the predicted incidence at the municipality level:*

collapse (sum) inc_pred h_inc, by(municipality_code municipality province ddcoordx ddcoordy)

** 4) Expressing the result in 10,000 person-year:*

replace inc_pred =inc_pred*10000

** 5) Display the annual mean incidence rate in the predicted data:*

list inc_pred, mean(inc_pred)

** NOTE: This is the dataset used for mapping the incidence rates predicted by TR algorithm, as illustrated in Figure 6.*

**** Run gradient boosting (GB) predicted values:***

cap rm CV.dta

use scen1_train , clear

r_ml_stata_cv $y $X , mlmodel("boost") data_test("unlabeled_data") tree_depth(3) n_estimators(50) learning_rate(0.1) prediction("pred") cross_validation("CV") n_folds(5) seed(10)

ereturn list

** Recovery of the value of the incidence predicted by the model in its anti-logarithmic form.*

** 1) Delete the data from the* train_data *dataset:*

drop if _train_index == "train"

** 2) Generate a new variable with the antilog form of the predicted values:*

gen inc_pred = exp(pred)

** 3) Collapsing the predicted incidence at the municipality level:*

collapse (sum) inc_pred h_inc, by(municipality_code municipality province ddcoordx ddcoordy)

** 4) Expressing the result in 10,000 person-year:*

replace inc_pred =inc_pred*10000

** 5) Display the annual mean incidence rate in real and predicted data:*

list inc_pred, mean(inc_pred)

** NOTE: This is the dataset used for mapping the incidence rates predicted by GB algorithm, as illustrated in Figure 6.*
